# Association of Vitamin B12 Status with Polysomnographic Parameters and Cardiovascular Disease in Patients with Obstructive Sleep Apnoea

**DOI:** 10.3390/nu17193079

**Published:** 2025-09-27

**Authors:** Izolde Bouloukaki, Antonios Christodoulakis, Theofilos Vouis, Violeta Moniaki, Eleni Mavroudi, Eleftherios Kallergis, Ioanna Tsiligianni, Sophia E. Schiza

**Affiliations:** 1 Department of Social Medicine, School of Medicine, University of Crete, 71500 Heraklion, Greece; izolthi@gmail.com (I.B.); i.tsiligianni@uoc.gr (I.T.); 2 Sleep Disorders Center, Department of Respiratory Medicine, Medical School, University of Crete, 71500 Heraklion, Greece; theofvouis@gmail.com (T.V.); vmoniaki@yahoo.gr (V.M.); elenima23@hotmail.com (E.M.); schizas@uoc.gr (S.E.S.); 3 Cardiology Department, Heraklion University Hospital, 71500 Crete, Greece; ekallergis@med.uoc.gr; 4 Department of Social Sciences, University of Nicosia, 1700 Nicosia, Cyprus; 5 Department of Respiratory Medicine, School of Medicine, University of Crete, 70013 Heraklion, Greece

**Keywords:** obstructive sleep apnoea, Vitamin B12, objective sleep quality, polysomnography, cardiovascular disease

## Abstract

**Background**: There are limited data on the association between B12 levels, objective sleep quality, and cardiovascular disease in patients with obstructive sleep apnoea (OSA). Therefore, the aim of our study was to assess vitamin B12 levels in a sleep clinic population in Crete, Greece, and investigate possible correlations with polysomnographic parameters and prevalent cardiovascular disease (CVD). **Methods**: In this cross-sectional study, data from 1468 recruited patients with OSA from the clinical database of the Sleep Disorders Center, Department of Respiratory Medicine, School of Medicine, University of Crete, were analyzed. OSA was defined as an apnoea–hypopnoea index ≥ 5 events per hour of sleep after type-1 Polysomnography (PSG). Data regarding anthropometrics, socio-demographics, and medical history was obtained. Logistic regression analysis was applied to examine the effect of vitamin B12 levels on PSG parameters and prevalent CVD after controlling for potential explanatory variables, including age, gender, obesity, smoking status, and co-morbidities. **Results**: The median vitamin B12 was 380.5 (301, 490) pg/mL. After adjustments, Vitamin B12 levels < 380.5 were associated with 24% higher odds of prolonged sleep latency (≥40 min) prevalence (OR = 1.240, 95% CI = 1.005–1.531, *p* = 0.045) and alterations in the proportion of NREM and REM sleep stages with 2.3 times higher likelihood of elevated NREM sleep > 80% of total sleep time (OR = 2.312, 95% CI = 1.049–5.096, *p* = 0.038) and 2.9 times higher likelihood of low REM sleep < 20% of total sleep time (OR = 2.858, 95% CI = 1.197–6.827, *p* = 0.018). Moreover, Vitamin levels < 380.5 were significantly associated with a 59.9% increase in the odds of prevalent CVD (OR = 1.599, 95% CI = 1.035–2.471, *p* = 0.034). **Conclusions**: In conclusion, our results suggest that vitamin B12 status may be associated with impaired objective sleep quality in OSA patients, potentially influencing prevalent CVD. However, further prospective research is needed to establish causality and elucidate the potential underlying mechanisms that could link vitamin B12 levels to various sleep parameters and cardiovascular disease in patients with OSA.

## 1. Introduction

Obstructive Sleep Apnoea (OSA) is a multifactorial disease characterized by repeated episodes of partial or complete upper airway obstruction during sleep, resulting in intermitted hypoxia, arousals, sleep fragmentation and autonomic dysfunction [1]. The global prevalence of OSA varies widely across studies, from 9% to 37% in men and 4% to 50% in women [2]. Population-based studies, however, consistently demonstrate a substantial high prevalence of undiagnosed OSA [3].

Untreated OSA results in adverse outcomes such as cardiovascular and metabolic diseases, depression, and exacerbation of comorbid diseases, placing a significant strain on healthcare resources [4]. These outcomes are associated with various biological pathways, including chronic intermittent hypoxemia, sleep fragmentation, hemodynamic disturbances, and autonomic alterations [5]. The intermittent hypoxia leads to inflammation, oxidative stress, overproduction of reactive oxygen species (ROS), resulting in endothelial dysfunction which is a significant contributor to cardio-metabolic diseases [6,7]. Within this context, the identification of antioxidant and anti-inflammatory agents as a complementary treatment strategy for patients with OSA has been investigated [8].

A number of vitamins demonstrate antioxidant and anti-inflammatory properties, with the potential to favorably impact OSA consequences and lessen the likelihood of cardiovascular complications [9]. Available studies, while often limited by cross-sectional designs and small sample sizes, indicate that vitamin deficiencies may play a role in the severity, cardiovascular and metabolic complications of OSA [9]. Among vitamins A, B, C, D, and E, which exhibit antioxidant properties, vitamin D has been the subject of the majority of studies; decreased serum vitamin D levels positively correlate with increased OSA severity [10]. The most limited data are for B-complex vitamins, especially vitamin B12 [11,12,13,14,15]. Vitamin B12, a critical nutrient involved in various physiological processes, including nerve function and red blood cell formation, has recently attracted attention for its potential link to sleep disorders [16,17,18,19,20]. This could be since Vitamin B12 is closely related to melatonin synthesis, the hormone responsible for regulating circadian rhythms and sleep–wake cycles [21,22,23,24,25]. Nevertheless, the majority of studies have primarily concentrated on the association between vitamin B12 and the duration of sleep and insomnia symptoms, without reaching definitive conclusions [25,26,27,28,29,30]. In addition, some studies relied on dietary intake assessments to determine vitamin B12 levels [29,31]. In this case, participants may have inaccurately recalled their intake, while dietary assessment tools themselves, such as food frequency questionnaires or diet diaries, are prone to error. This makes it difficult to accurately estimate vitamin B12 intake, and could compromise the validity of these findings. However, overall, there is a scarcity of information regarding the impact of B12 levels on OSA severity [11,12,32,33,34], objective sleep quality and cardiovascular disease.

Given the aforementioned findings, further study is needed, especially in populations where data is lacking, like Greece. While the traditional Greek diet typically includes adequate Vitamin B12 intake, dietary shifts may place specific groups, such as vegans or individuals with limited consumption of meat, fish, and dairy at risk, necessitating consideration of supplements or fortified foods [35]. Therefore, we hypothesized that vitamin B12 levels could influence objective sleep parameters and/or cardiovascular disease. To test this hypothesis, we assessed serum vitamin B12 levels in a sleep clinic population in Crete, Greece, and examined the potential associations with polysomnography (PSG) parameters, and prevalent cardiovascular disease by also taking into account other confounders.

## 2. Materials and Methods

### 2.1. Study Design

In this single-center, cross-sectional study of prospectively enrolled patients, consecutive patients aged ≥18 years were evaluated in our Sleep Disorders Center for suspected sleep-disordered breathing during a 7-year period (2018–2024).

### 2.2. Participants

**Figure 1:**
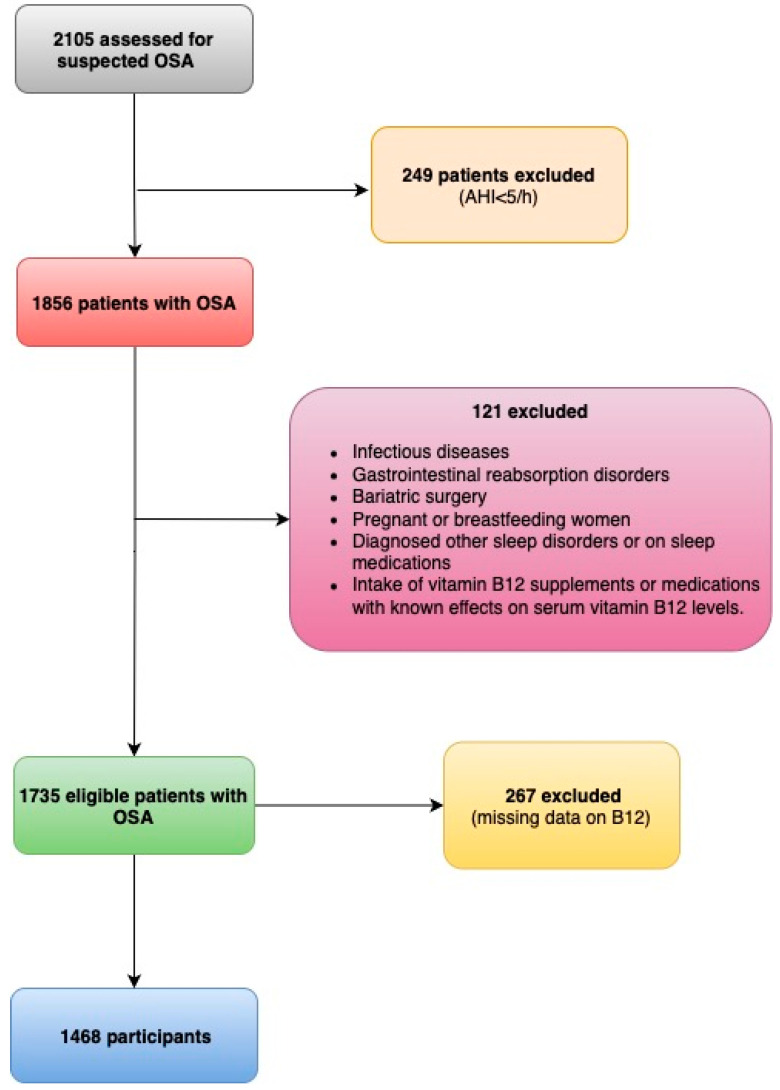
The flowchart of patients finally included.

Initially, 2105 adult patients (≥18 years) suspected of having OSA were evaluated. This study included participants with an apnoea–hypopnoea index (AHI) of at least 5 events per hour during polysomnography (PSG), complete vitamin B12 level data, and questionnaires’ scores, were included. Exclusion criteria were incomplete data; vitamin B12 supplement/medication use (e.g., metformin, PPIs) within six months; other sleep disorders (e.g., narcolepsy); and conditions potentially impacting vitamin B12 absorption (e.g., pernicious anemia). Trained personnel systematically reviewed the database to identify and contact eligible patients for participation. The review/selection process is further depicted in Figure 1. After the initial review/selection, 1468 patients met the inclusion criteria and were included in the study.

### 2.3. Data Collection

Data collection entailed demographics (age, gender, etc.), body mass index (BMI), smoking status, presence of co-morbidities, PSG-related sleep parameters, and serum vitamin B12 levels.

#### 2.3.1. Polysomnography (PSG)

All participants spent a night in our Sleep Disorders Center for a full diagnostic PSG study using the Alice 5 Diagnostics System (Respironics, Murrysville, PA, USA). This study followed standard protocols, monitoring the electroencephalogram (EEG) with frontal, central, and occipital derivations, as well as the electro-oculogram, electromyogram, airflow (using an oronasal thermistor and nasal air pressure transducer), chest and abdominal respiratory effort (via respiratory inductance plethysmography), pulse oximetry (SpO_2_), and body position. A microphone placed on the anterior neck recorded snoring. Apnoea and hypopnoea were defined by the American Academy of Sleep Medicine’s standard criteria [36]. The analysis included total sleep time (TST), sleep efficiency [SE (%)], wake after sleep onset (WASO), arousal index (AI), apnoea–hypopnoea index (AHI), oxygen desaturation index (ODI), resting room air pulse oximetry (SpO_2_), and sleep time with oxygen saturation < 90% (TST90). Sleep efficiency was defined as the percentage of time spent sleeping while in bed, and low sleep efficiency was defined as sleep efficiency ≤ 85%. Sleep latency was defined as the time (in minutes) taken to fall asleep at night; long sleep latency was categorized as >40 min. Long WASO was defined as ≥50 min. Low REM and N3 sleep was defined as less than 20% of TST, while high NREM sleep was defined as over 80% of TST [37]. The severity of OSA was categorized based on the AHI as follows: mild (5 to <15 events per hour), moderate (15 to <30 events per hour), and severe (≥30 events per hour). Only participants with an AHI of at least 5 events per hour were included in our study.

#### 2.3.2. Vitamin B12 Levels

Following an 8 h fast, blood samples were collected, centrifuged (3000 rpm, 10 min), and stored frozen at −80 °C until further analysis. Serum vitamin B12 levels were measured using the Alinityi system (Abbott Laboratories, Chicago, IL, USA) chemiluminescent microparticle immunoassay (CMIA) analyzer. As different cut-off values for B12 levels have been proposed in various populations and there is no universally accepted value to define the “deficiency” or “sufficiency” of B12 [38,39], this study used the median serum vitamin B12 level to describe the typical B12 status within the population studied. Then participants were split into two groups according to whether their vitamin B12 levels were below (considered low) or at or above (considered normal) the median value.

### 2.4. Statistical Analysis

Continuous data, if normally distributed, are presented as mean ± standard deviation; otherwise, they are presented as median (25th–75th percentile). Qualitative data are shown as *n* (%). For group comparisons, we used independent samples t-tests (normally distributed data) or Mann–Whitney U-tests (non-normally distributed data) for continuous variables, and Chi-square tests for categorical variables. Logistic stepwise multiple regression procedures were used to determine influencing PSG parameters of low vitamin B12 levels (below median value) and relevant influencing factors of prevalent cardiovascular disease, after adjusting for age, sex, BMI, smoking, comorbidities, and OSA severity. Collinearity diagnostics (tolerance and variance inflation factor) ensured acceptable multicollinearity among predictors. Cardiovascular disease included coronary artery disease, atrial fibrillation, cerebrovascular disease, and heart failure. Age (18–59 vs. >60 years) and BMI (<30 vs. ≥30 kg/m^2^) were analyzed both continuously and categorically. Significance was set at *p* < 0.05. Analyses were performed using SPSS version 25 (SPSS Inc., Chicago, IL, USA).

## 3. Results

### 3.1. Study Population

After the screening, 1468 participants were eligible and enrolled in this study (average age 54, 73% male), as shown in Figure 1. The median level of vitamin B12 was 380.5 (301, 490) pg/mL, and there was no statistically significant difference observed between genders (male: 377 vs. female: 389, *p* = 0.058). Five percent of the participants had Vitamin B12 levels below 200 pg/mL. Participants were split into two groups according to whether their vitamin B12 levels were below (low: <380.5 pg/mL) or at or above (normal: ≥380.5 pg/mL) the median value of 380.5 pg/mL.

Table 1 presents the baseline characteristics of the study participants, categorized by their vitamin B12 status. Those with low vitamin B12 levels showed no significant differences in baseline characteristics compared to those with normal levels. This included factors like gender, BMI, existing health conditions, and smoking status (all *p* > 0.05), with the exception of age (55 vs. 53, *p* = 0.028).

### 3.2. Vitamin B12 Correlations with PSG Parameters

Table 2 shows comparisons of various PSG parameters between participants with low vitamin B12 levels and those without. Compared with patients with vitamin B12 levels ≥ 380.5, those with levels < 380.5 pg/mL showed a small increase in the likelihood to experience impairment in sleep quality with lower Sleep efficiency (%) (<380.55 pg/mL: 64 vs. ≥380.5 pg/mL: 66, *p* = 0.019) and REM (%TST) sleep (<380.5 pg/mL: 9 vs. ≥380.5 pg/mL: 10, *p* = 0.031), and higher sleep latency (<380.5 pg/mL: 41 vs. ≥380.5 pg/mL: 38, *p* = 0.008), especially sleep latency > 40 min (<380.5 pg/mL: 52 vs. ≥380.5 pg/mL 47%, *p* = 0.028). Higher NREM sleep, whereas lower REM sleep were more prevalent in patients with low levels of Vitamin B12 (<380.5 pg/mL: 98 vs. ≥380.5 pg/mL: 96%, *p* = 0.03, and ≥380.5 pg/mL: 96 vs. <380.5 pg/mL: 98%, *p* = 0.014, respectively).

No differences were observed in other sleep quality parameters [WASO, SWS (%TST), Arousal Index] or OSA severity indices [AHI, AHI REM, ODI, Mean and Lowest SpO_2_, TST90 (%)].

Logistic regression analysis, which explored the association between serum B12 status and PSG parameters (Table 3), showed that vitamin B12 < 380.5 pg/mL was positively associated with higher sleep latency (OR = 1.006, 95% CI = 1.003–1.009, *p* < 0.001) and with 24% higher odds of prolonged sleep latency (≥40 min) prevalence (OR = 1.240, 95% CI = 1.005–1.531, *p* = 0.045). This means that these patients with low Vitamin B12 are more prone to falling asleep later than usual. Moreover, Vitamin B12 < 380.5 pg/mL was associated with 2.3 times higher likelihood of high NREM sleep (OR = 2.312, 95% CI = 1.049–5.096, *p* = 0.038), was, inversely associated with REM (%TST) sleep (OR = 0.972, 95% CI = 0.944–1.000, *p* = 0.049) and 2.9 times higher likelihood of low REM sleep (OR = 2.858, 95% CI = 1.197–6.827, *p* = 0.018). This pattern in patients with low Vitamin B12, characterized by an alteration in the proportion of NREM and REM sleep stages, suggests a possible imbalance or disturbance in the natural sleep cycle.

### 3.3. Vitamin B12 Correlations with Prevalent CVD

Table 4 presents a multiple stepwise logistic regression analysis examining the relationship between CVD and various independent variables. Vitamin levels < 380.5 pg/mL were significantly associated with a 59.9% increase in the odds of prevalent CVD after adjustment for confounders (OR = 1.599, 95% CI = 1.035–2.471, *p* = 0.034). Age ≥ 60 years, presence of hypertension, type 2 diabetes, hyperlipidemia and COPD were also associated with increased odds for CVD.

## 4. Discussion

The present cross-sectional study explored the association between vitamin B12 levels, PSG parameters, and presence of cardiovascular disease in 1468 patients with OSA. Our findings revealed that vitamin B12 levels below the median (<380 pg/mL) was associated with impaired objective sleep quality, characterized by longer sleep onset latency, longer NREM sleep, and reduced REM sleep. Furthermore, a significant positive association was noted between vitamin B12 levels below the median and a greater prevalence of cardiovascular disease.

Vitamin B12 serves as an essential nutrient for normal nervous system function, maintaining myelin sheath integrity, and supporting neurotransmitter production [40]. While the neurobiological mechanisms underlying Vitamin B12’s effects on sleep remain unclear, studies have suggested that Vitamin B12 may impact the synthesis of sleep-regulating neurotransmitter melatonin [21]. This finding raises the possibility that vitamin B12, through its influence on melatonin, could disrupt normal sleep patterns and potentially causing sleep disturbances. In our study we found that vitamin B12 levels < 380.5 pg/mL were associated with longer sleep onset latency and NREM sleep as well as with reduced REM sleep, thus suggesting worse objective sleep quality. However, there has been no prior research utilizing PSG studies with objective sleep quality measures to explore their association with B12 status, and existing evidence is based on subjective evaluations of sleep quality. A previous study using subjective sleep quality measures showed the reverse effect—higher serum vitamin B12 levels correlated with shorter sleep duration and better scores for the sleep quality components in 355 female Arab students, supporting our findings [27]. Furthermore, one study reported that insufficient vitamin B12 intake was associated with increased sleep latency in a group of 112 young Japanese women [29]. In contrast, other research found no link between vitamin B12 levels and sleep quality, based on the subjective Pittsburgh Sleep Quality Index [22,37]. Variations in study methodologies, including subjective versus objective sleep quality measures, diverse study populations (lack of focus on OSA patients), and differences in serum vitamin B12 levels and sleep durations across racial and ethnic groups, may account for these inconsistencies [39,41].

The existing data on objective sleep quality and its relationship to vitamin B12 status are scarce [31,42]. A prior study of 14 healthy adults, using actigraphy to track sleep for four weeks (two weeks pre- and two weeks post-supplementation with 3 mg/day cyanocobalamin), found no correlation between pre- and post-supplementation vitamin B12 levels and sleep parameters (sleep latency, WASO, total sleep time, total time in bed, sleep efficiency) [42]. In a study of 34 elite female athletes, actigraphy and sleep diaries (10-day period) assessed the relationship between dietary vitamin B12 and sleep parameters. Findings revealed a significant association between vitamin B12 intake and lower WASO, as well as increased sleep efficiency [31]. Other relevant studies only compare B12 levels in control subjects and OSA patients of varying severity, without exploring associations between B12 and objective sleep quality measures [11,12,32,33,34]. These studies found no association between Vitamin B12 status and OSA severity, which is also the case in our study.

Another important finding of our study was that individuals with OSA and vitamin B12 levels below the median exhibited a 1.6 times higher likelihood of prevalent cardiovascular disease. Evidence suggests low vitamin B12 levels may accelerate and worsen cardiovascular disease via various molecular mechanisms, such as chronic inflammation and oxidative stress [43]. Homocysteine accumulation, potentially resulting from insufficient vitamin B12 which is crucial for its metabolism, may partly explain the observed increase in oxidative stress [16,44]. Therefore, the association of lower B12 levels with oxidative stress and reduced antioxidant capacity [16], coupled with the oxidative stress induced by OSA [6], indicates an additive effect on cardiovascular disease presence when both conditions are present. However, further research is needed to validate these findings.

Our findings have significant implications for clinical practice and future studies. Individuals with vitamin B12 levels < 380.5 pg/mL experienced poorer objective sleep quality, which suggests that increasing B12 intake through diet or supplements might improve sleep quality. The aforementioned findings highlight the importance of using objective (polysomnography) for a more accurate evaluation of sleep quality when making treatment decisions. It is also of critical importance to identify OSA patients with vitamin B12 levels below the median, as this group could be associated with a heightened risk of cardiovascular disease. This evidence underscores the potential of vitamins and nutritional interventions as adjunctive therapies for OSA, complementing established treatments such as continuous positive airway pressure (CPAP), cognitive behavioral therapy, positional therapy, and lifestyle modifications [45]. Furthermore, clinicians could potentially utilize serum vitamin B12 measurements to predict the onset of cardiovascular disease, and adapt the therapies focused on adequate B12 intake and oxidative stress. Future research could explore whether structured diets, particularly those rich in nutrients, such as vitamin B12, can improve symptoms, sleep quality, and cardiovascular health in patients with OSA.

### Strengths and Limitations

To the best of our knowledge, this is the first study to evaluate serum vitamin B12 levels and their associations with objective sleep quality measures and the presence of cardiovascular disease within a Greek sleep clinic population. This study’s strengths include a large sample size (*n* = 1468) and the use of type-1 polysomnography for estimating objective sleep quality. However, there are some limitations that deserve comment. First, due to the cross-sectional design of the study, it is not possible to determine a clear cause-and-effect or pathophysiologic association between the identified predictors and vitamin B12 concentrations. Nevertheless, given the scarcity of relevant studies in the literature, this research is valuable as a starting point for future prospective and interventional studies. Second, the median-based vitamin B12 cutoff (380.5 pg/mL) may not represent clinically significant low levels, but as there is no universally accepted value to define the “deficiency” or “sufficiency” of B12 we used the median serum vitamin B12 level to split the population into two groups according to whether their vitamin B12 levels were below or at or above the typical B12 status within the population studied. Given that a population-based median threshold serves as a population-specific point of reference, a comparison with established clinical cutoffs for vitamin B12 deficiency, such as less than 200 pg/mL, would be valuable. Such a comparison can guide clinicians in understanding if the population is at risk overall and if focused interventions are warranted.

Third, a single vitamin B12 measurement may not represent functional markers of vitamin B12 adequacy, such as serum total homocysteine and methylmalonic acid [46]. Furthermore, although we controlled for many important confounding variables, unmeasured factors like diet, eating habits, exercise and stress could still affect our findings. However, given that our study included only Cretans from the same region with similar diets, we anticipate no substantial dietary differences. Finally, given that the research involved OSA patients exclusively, without healthy controls, and that the participants resided in Crete, a southern Greek region, the extrapolation of our findings to general population and all Greek patients with OSA warrants caution.

## 5. Conclusions

In conclusion, our findings indicate that vitamin B12 levels <380.5 pg/mL are associated with worse objective sleep quality and with increased cardiovascular disease prevalence. These findings suggest that vitamin B12 status may be associated with impaired sleep quality in OSA patients and potentially predicting presence of cardiovascular disease. This observation underscores the potential significance of assessing and addressing vitamin B12 levels as an integral component of a comprehensive approach and as “adjunctive approach” to enhancing sleep and cardiovascular health within this patient population. However, additional research is warranted to determine if there is a causal relationship and to investigate the potential biological pathways that connect vitamin B12 levels with sleep quality and cardiovascular health in patients with OSA.

## Figures and Tables

**Table 1:** Clinical characteristics of patients based on vitamin B12 status.

		Total Population According to Vitamin B12 Status		
	Total Population	Vitamin B12 ≥ 380.5 pg/mL	Vitamin B12 < 380.5 pg/mL	*p*-Value
	*N* = 1468	*N* = 734	*N* = 734	
**Demographics**				
Gender, males (%)	1070 (73%)	523 (71%)	547 (75%)	0.159
Age, years	53.92 ± 14.47	53.08 ± 14.33	54.75 ± 14.57	0.028
BMI (kg/m^2^)	33.02 ± 7.25	33.04 ± 7.29	33.00 ± 7.22	0.908
Current smoking, *n* (%)	400 (27%)	206 (28%)	194 (26%)	0.729
**Co-morbidities**				
Hypertension	617 (42%)	294 (40%)	323 (44%)	0.125
Cardiovascular disease	240 (16%)	109 (15%)	131 (18%)	0.118
Diabetes Type 2	239 (16%)	111 (15%)	128 (18%)	0.234
Hyperlipidemia	600 (41%)	300 (41%)	300 (41%)	0.983
COPD	186 (13%)	94 (13%)	92 (13%)	0.875
Asthma	125 (9%)	63 (9%)	62 (9%)	0.919
Depression (on medication)	171 (12%)	84 (12%)	87 (12%)	0.807

Data are presented as mean values  ±  SD or median (25th–75th percentile), unless otherwise indicated. BMI, body mass index; COPD, chronic obstructive pulmonary disease; cardiovascular disease: coronary heart disease or atrial fibrillation or cerebrovascular disease or heart failure.

**Table 2:** PSG parameters of patients according to vitamin B12 status.

		Total Population According to Vitamin B12 Status		
	Total Population	Vitamin B12 ≥ 380.5 pg/mL	Vitamin B12 < 380.5 pg/mL	*p*-Value
	*N* = 1468	*N* = 734	*N* = 734	
**Sleep efficiency (%)**	65 ± 13	66 ± 12	64 ± 13	0.019 *
**Sleep efficiency < 85% (%)**	1404 (96%)	703 (96%)	701 (96%)	0.793
**WASO**	108 ± 48	106 ± 48	110 ± 48	0.126
**WASO ≥ 50 min (%)**	1324 (90%)	663 (90%)	661 (90%)	0.782
**Sleep latency**	39 (26, 62)	38 (25, 58)	41 (26, 68)	0.008 *
**Sleep latency > 40 min (%)**	727 (50%)	342 (47%)	385 (52%)	0.028 *
**NREM (%TST)**	90 ± 4	89 ± 4	90 ± 4	0.060
**NREM > 80%TST (%)**	1423 (97%)	703 (96%)	720 (98%)	0.030 *
**SWS (%TST)**	6 (6, 9)	7 (5, 9)	6 (5, 8)	0.267
**SWS < 20%TST (%)**	1421 (97%)	706 (96%)	721 (98%)	0.150
**REM (%TST)**	9 ± 4	10 ± 4	9 ± 4	0.031 *
**REM < 20%TST (%)**	1421 (97%)	701 (96%)	720 (98%)	0.014 *
**Arousal index**	42 ± 16	43 ± 16	42 ± 15	0.450
**AHI**	32 (17, 60)	32 (18, 62)	32 (17, 57)	0.298
**AHI REM**	39 (20, 63)	41 (21, 66)	39 (20, 60)	0.106
**ODI**	34 (17, 61)	34 (17, 63)	33 (17, 59)	0.389
**Mean SpO_2_**	93 (90, 94)	93 (89, 94)	93 (90, 94)	0.930
**Lowest SpO_2_**	81 (77, 86)	81 (77, 85)	81 (77, 86)	0.259
**TST90 (min)**	45 (12, 102)	46 (13, 102)	44 (11, 102)	0.351

* Indicates a *p* < 0.05. Data are presented as mean values ± SD or median (25th–75th percentile), unless otherwise indicated. AHI: apnoea–hypopnoea index; ODI: oxygen desaturation index; SpO_2_: resting room air pulse oximetry; TST: Total sleep time; TST90: sleep time with oxygen saturation < 90%; WASO: wakefulness after sleep onset.

**Table 3:** Multiple stepwise logistic regression analysis of the relationship between Vitamin B12 status (<380.5) and various PSG parameters adjusted for age, sex, BMI, smoking, comorbidities, and OSA severity.

	B	S.E.	*p*-Value	OR (95%CI)
**Sleep latency (min)**	0.006	0.002	<0.001	1.006 (1.003–1.009)
**Sleep latency ≥ 40 min**	0.215	0.107	0.045	1.240 (1.005–1.531)
**NREM > 80%TST**	0.838	0.403	0.038	2.312 (1.049–5.096)
**REM (%TST)**	−0.029	0.015	0.049	0.972 (0.944–1.000)
**REM < 20%TST**	1.050	0.444	0.018	2.858 (1.197–6.827)

AHI: apnoea–hypopnoea index; ODI: oxygen desaturation index; SpO_2_: resting room air pulse oximetry; TST: Total sleep time; TST90: sleep time with oxygen saturation < 90%; WASO: wakefulness after sleep onset.

**Table 4:** Multiple stepwise logistic regression analysis between prevalent CVD and various independent variables.

	B	S.E.	*p*-Value	OR (95%CI)
**Males versus females**	0.393	0.203	0.053	1.481 (0.995–2.203)
**Age ≥ 60 years**	1.036	0.239	<0.001	2.817 (1.763–4.562)
**Body mass index ≥ 30**	0.274	0.187	0.144	1.315 (0.911–1.897)
**Current/former smoking**	0.003	0.187	0.988	1.003 (0.695–1.447)
**Hypertension**	0.401	0.176	0.023	1.494 (1.058–2.109)
**Diabetes type 2**	0.589	0.181	0.001	1.801 (1.263–2.570)
**Hyperlipidemia**	0.443	0.162	0.006	1.558 (1.134–2.141)
**COPD**	0.496	0.206	0.016	1.642 (1.097–2.457)
**Depression**	0.065	0.238	0.783	1.068 (0.670–1.701)
**Inflammatory Arthritis**	0.053	0.303	0.860	1.055 (0.583–1.909)
**Asthma**	−0.185	0.294	0.529	0.831 (0.467–1.479)
**Vitamin B12 < 380.5**	0.469	0.222	0.034	1.599 (1.035–2.471)
**AHI ≥ 15/h**	0.546	0.280	0.051	1.727 (0.998–2.987)

AHI: Apnoea–hypopnoea index; COPD: chronic obstructive pulmonary disease.

## Data Availability

The datasets generated during and analyzed during the current study are available from the corresponding author on reasonable request.
